# Necrolytic migratory erythema associated with painful plantar keratoderma. A new diagnostic clue for this paraneoplastic syndrome?^☆^^☆☆^

**DOI:** 10.1016/j.abd.2020.02.011

**Published:** 2020-07-15

**Authors:** Inés Gracia-Darder, Daniel Ramos, Julián Boix-Vilanova, Ana Francisca Bauzá-Alonso

**Affiliations:** Department of Dermatology, Son Espases University Hospital, Palma de Mallorca, Baleares, Spain

Dear Editor,

The presence of recurrent skin lesion outbreaks in intertriginous areas and lower extremities, during years of evolution, may be a presenting form of necrolytic migratory erythema (NME), which is a paraneoplastic skin disease that is associated with malignant glucagonoma in 90% of cases.^1^

This report presents the case of a 59-year-old woman with a history of keratoconjunctivitis, asthenia, and constipation. She was referred to evaluate recurrent skin lesions with four years of evolution. She was affected by erythematous, scaly plaques and hyperpigmentation in the legs, gluteal area, groin area, thighs, and elbows, with no associated systemic symptoms (Fig. 1). She provided several skin biopsies, which had been diagnosed as toxicoderma and eczema, but the patient denied taking drugs. A new biopsy showed an epidermis with a marked pale central area due to the presence of apoptotic keratinocytes of vacuolated appearance below a layer of extensive hyperkeratosis and parakeratosis (Fig. 2). This gave a “tricolor flag” image, which is suggestive of vitamin deficiency. In addition, a deficit of zinc and fatty acids was observed, and, after starting vitamin and zinc supplements, the patient remained asymptomatic for eight months. Nevertheless, she subsequently presented more severe outbreaks with blisters, edema, and scaling predominantly on the dorsum of the feet, associated with a very painful plantar keratoderma (Fig. 3).

**Figure 1:**
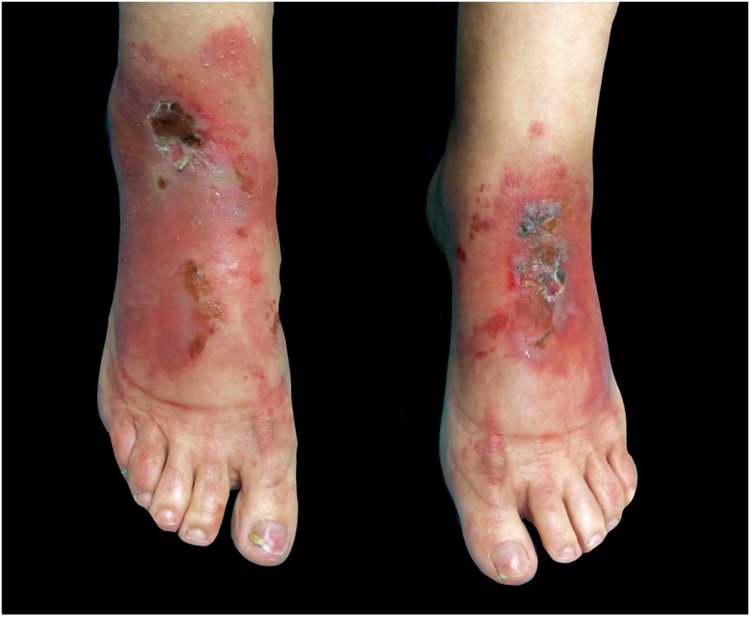
Erythematous, scaly, erosive, and crusty lesions on the dorsum of the feet.

**Figure 2:**
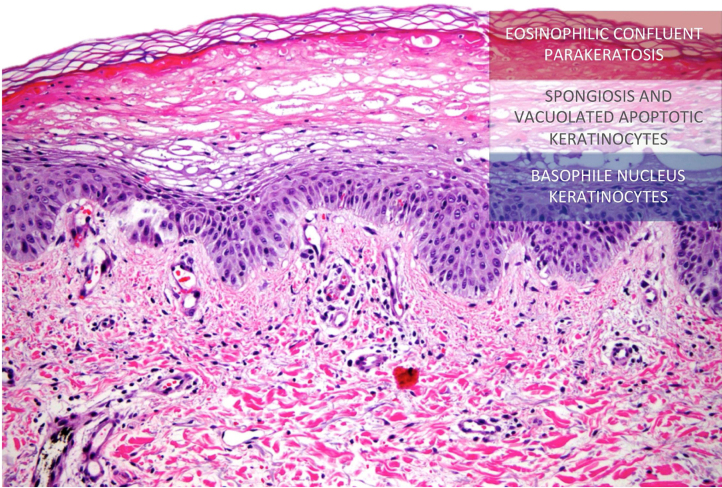
Histology of the lesions. Atrophic epidermis can be seen with moderate spongiosis and a central area with marked pallor due to the presence of apoptotic keratinocytes of vacuolated appearance below a more superficial layer of extensive hyperkeratosis and parakeratosis, producing a “tricolor flag” appearance (Hematoxylin & eosin, ×20).

**Figure 3:**
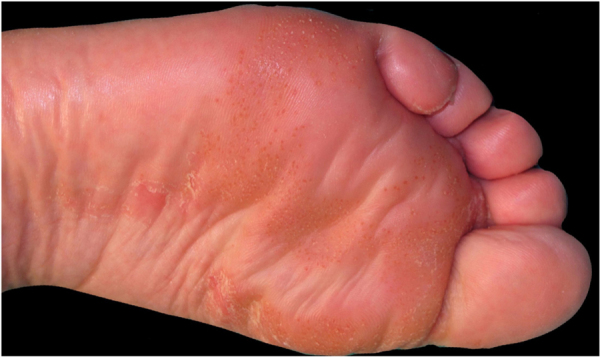
Plantar hyperkeratotic patches with reddish dots.

With the suspicion of NME and high levels of glucagon, an abdominal CT scan was made showing a 4 cm mass in the pancreas, and the presence of a malignant glucagonoma was confirmed by pathological anatomy. The skin lesions completely resolved after tumor resection. However, one year later she developed liver metastasis with no recurrence of skin lesions.

Although malignant glucagonoma may be accompanied by systemic symptoms such as diarrhea, weight loss, newly developed diabetes, normocytic anemia, zinc deficiency, or fatty acid or amino acid deficiency, *etc.*, NME may be the first and only glucagonoma symptom.^2^, ^3^ The pathogenesis of NME can be explained due to the fact that hyperglucagonemia stimulates hepatocyte gluconeogenesis and lipolysis leading to hypoaminoacidemia. Liver dysfunction results in decreased albumin, which is the main carrier of zinc and fatty acids, and thus contributes to fatty acid and zinc deficiency. Glucagon also causes vitamin B and nutrient deficiencies, such as zinc deficiency, which may contribute to increased levels of arachidonic acid, prostaglandins, and leukotrienes, and thus predisposing individuals to develop inflammatory skin lesions, such as NME, and the classic epidermal necrolysis seen in the histology.^1^ The evolution of skin lesions in outbreaks, which are sometimes self-healing, the nonspecific histology in some cases, and the long evolution of the lesions are the reason for the diagnostic delay.^3^ It should be noted that the “tricolor flag” histological image is associated not only with skin lesions due to nutritional deficits and acral necrolytic erythema, but also with the advanced cutaneous lesions of NME.^4^ It is also important to highlight the presence of a painful plantar keratoderma, which was not found to be associated with this syndrome in the literature, and which was completely resolved with the removal of the neoplasm. Even though malignant glucagonoma is a slow-growing tumor, more than 50% of the cases at diagnosis already have metastatic involvement.^5^ For this reason, it is crucial to highlight the importance of an early diagnosis of this clinical presentation, which can lead to preventing the appearance of metastases leading to a worse prognosis. In conclusion, this report describes patient with recurrent skin lesions, where the histological findings were the key to establishing the diagnosis, as the only manifestation of a malignant glucagonoma.

## Financial support

None declared.

## Authors' contributions

Inés Gracia Darder: Approval of the final version of the manuscript; conception and planning of the study; drafting and editing of the manuscript; collection, analysis, and interpretation of data; participation in study design; intellectual participation in the propaedeutic and/or therapeutic conduct of the studied cases; critical review of the literature; critical review of the manuscript.

Daniel Ramos: Approval of the final version of the manuscript; conception and planning of the study; drafting and editing of the manuscript; collection, analysis, and interpretation of data; participation in study design; intellectual participation in the propaedeutic and/or therapeutic conduct of the studied cases; critical review of the literature; critical review of the manuscript.

Julián Boix Vilanova: Approval of the final version of the manuscript; conception and planning of the study; drafting and editing of the manuscript; collection, analysis, and interpretation of data; participation in study design; intellectual participation in the propaedeutic and/or therapeutic conduct of the studied cases; critical review of the literature; critical review of the manuscript.

Ana Francisca Bauzá Alonso: Approval of the final version of the manuscript; conception and planning of the study; drafting and editing of the manuscript; collection, analysis, and interpretation of data; participation in study design; intellectual participation in the propaedeutic and/or therapeutic conduct of the studied cases; critical review of the literature; critical review of the manuscript.

## Conflicts of interest

None declared.
